# Markers of Inflammation and Coagulation after Long-Term Exposure to Coarse Particulate Matter: A Cross-Sectional Analysis from the Multi-Ethnic Study of Atherosclerosis

**DOI:** 10.1289/ehp.1308069

**Published:** 2015-01-20

**Authors:** Sara D. Adar, Jennifer D’Souza, Kari Mendelsohn-Victor, David R. Jacobs, Mary Cushman, Lianne Sheppard, Peter S. Thorne, Gregory L. Burke, Martha L. Daviglus, Adam A. Szpiro, Ana V. Diez Roux, Joel D. Kaufman, Timothy V. Larson

**Affiliations:** 1 Department of Epidemiology, University of Michigan, Ann Arbor, Michigan, USA; 2 Division of Epidemiology and Community Health, University of Minnesota, Minneapolis, Minnesota, USA; 3 Department of Medicine, and; 4 Department of Pathology, University of Vermont, Burlington, Vermont, USA; 5 Department of Environmental and Occupational Health Sciences, and; 6 Department of Biostatistics, University of Washington, Seattle, Washington, USA; 7 Department of Occupational and Environmental Health, University of Iowa, Iowa City, Iowa, USA; 8 Division of Public Health Sciences, Wake Forest School of Medicine, Winston-Salem, North Carolina, USA; 9 Institute for Minority Health Research, University of Illinois at Chicago, Chicago, Illinois, USA; 10 Department of Preventive Medicine, Northwestern University, Chicago, Illinois, USA; 11 Department of Epidemiology,; 12 Department of Medicine, and; 13 Department of Civil and Environmental Engineering, University of Washington, Seattle, Washington, USA

## Abstract

**Background:**

Toxicological research suggests that coarse particles (PM_10–2.5_) are inflammatory, but responses are complex and may be best summarized by multiple inflammatory markers. Few human studies have investigated associations with PM_10–2.5_ and, of those, none have explored long-term exposures. Here we examine long-term associations with inflammation and coagulation in the Multi-Ethnic Study of Atherosclerosis.

**Methods:**

Participants included 3,295 adults (45–84 years of age) from three metropolitan areas. Site-specific spatial models were used to estimate 5-year concentrations of PM_10–2.5_ mass and copper, zinc, phosphorus, silicon, and endotoxin found in PM_10–2.5_. Outcomes included interleukin-6, C-reactive protein, fibrinogen, total homocysteine, D-dimer, factor VIII, plasmin–antiplasmin complex, and inflammation and coagulation scores. We used multivariable regression with multiply imputed data to estimate associations while controlling for potential confounders, including co-pollutants such as fine particulate matter.

**Results:**

Some limited evidence was found of relationships between inflammation and coagulation and PM_10–2.5_. Endotoxin was the PM_10–2.5_ component most strongly associated with inflammation, with an interquartile range (IQR) increase (0.08 EU/m^3^) associated with 0.15 (95% CI: 0.01, 0.28; *p* = 0.03) and 0.08 (95% CI: –0.07, 0.23; *p* = 0.28) higher inflammation scores before and after control for city, respectively. Copper was the component with the strongest association with coagulation, with a 4-ng/m^3^ increase associated with 0.19 (95% CI: 0.08, 0.30; *p* = 0.0008) and 0.12 (95% CI: –0.05, 0.30; *p* = 0.16) unit higher coagulation scores before and after city adjustment, respectively.

**Conclusions:**

Our cross-sectional analysis provided some evidence that long-term PM_10–2.5_ exposure was associated with inflammation and coagulation, but associations were modest and depended on particle composition.

**Citation:**

Adar SD, D’Souza J, Mendelsohn-Victor K, Jacobs DR Jr, Cushman M, Sheppard L, Thorne PS, Burke GL, Daviglus ML, Szpiro AA, Diez Roux AV, Kaufman JD, Larson TV. 2015. Markers of inflammation and coagulation after long-term exposure to coarse particulate matter: a cross-sectional analysis from the Multi-Ethnic Study of Atherosclerosis. Environ Health Perspect 123:541–548; http://dx.doi.org/10.1289/ehp.1308069

## Introduction

Recent estimates suggest that ambient particulate matter exposures result in nearly 3.5 million deaths and 76 million disability-adjusted life years lost globally each year (Lim et al. 2013). Increased morbidity and mortality from particulate matter (PM) is hypothesized to be caused partly by systemic inflammation and a hypercoagulable state following pulmonary oxidative stress and inflammation (Seaton et al. 1995). However, previously reported associations for airborne particles with inflammation and hypercoagulability are highly heterogeneous, with variations in the magnitude of these associations by personal characteristics as well as particle size and chemical composition (Gerlofs-Nijland et al. 2009; Halatek et al. 2011; Happo et al. 2010; Hetland et al. 2004; Monn and Becker 1999).

It has often been hypothesized that smaller particles (≤ 2.5 μm; PM_2.5_) have the greatest health impacts because they penetrate deep into the alveolar regions of the lung and are highly reactive (Brook et al. 2004). Although coarse particles (2.5–10 μm, PM_10–2.5_) deposit less in the alveolar regions of the lung and are often naturally occurring, they can still reach the lower airways and have high levels of particle-bound inflammatory biological material (U.S. Environmental Protection Agency 2009). In fact, numerous toxicological studies indicate that PM_10–2.5_ is more strongly associated with inflammation and coagulation than PM_2.5_. For example, PM_10–2.5_ was more strongly associated with *in vitro* cytokine production from human monocytes and alveolar macrophages than PM_2.5_ (Becker et al. 2003; Monn and Becker 1999). Similar results were found *in vivo* using bronchoalveolar lavage fluid collected from rodents (Happo et al. 2007, 2010; Schins et al. 2004; Tong et al. 2010). Relatively little, however, has been reported on relationships between PM_10–2.5_ and systemic inflammation as measured in blood, and research in humans is sparse. The few available epidemiology studies show some evidence of associations between short-term exposures and altered pulmonary cytokines, circulating cytokines, and circulating coagulation factors (Bonzini et al. 2010; Delfino et al. 2008; Graff et al. 2009; Peters et al. 2009; Yeatts et al. 2007) though results vary by biomarker and investigation.

Diversity in observed associations may reflect a multifaceted immune response that begins with a localized reaction including macrophage activation and ends with the release of cytokines systemically (Brook et al. 2010). Given this complexity, a summary metric of several concurrent pathways may better capture inflammatory burden than any single marker alone. Summary scores may also be a useful way to reflect the cumulative impact of long-term exposure to air pollution on inflammation and coagulation, which is a largely understudied area to date. Characterizing relationships between long-term exposures to particles, inflammation, and coagulation is important because it is one plausible mechanism underlying the observed associations of long-term particulate matter exposures with mortality (Adar et al. 2014; Dockery et al. 1993; Miller et al. 2007; Pope et al. 2002) and the development of atherosclerosis (Adar et al. 2013; Künzli et al. 2010).

To characterize associations with long-term exposures to PM_10–2.5_ mass and chemical components, we examined cross-sectional relationships with summary measures of inflammation and coagulation in the Multi-Ethnic Study of Atherosclerosis (MESA). Associations with PM_10–2.5_ endotoxin content—an innate immune modulating component of bacterial cell membranes—were similarly explored. We also assessed relationships with individual biomarkers [interleukin-6 (IL-6), C-reactive protein (CRP), fibrinogen, total homocysteine, factor VIII, D-dimer, and plasmin–antiplasmin complex (PAP)].

## Methods

*Study population*. MESA recruited 6,814 white, black, Hispanic, and Chinese participants from Baltimore, Maryland; Chicago, Illinois; Forsyth County, North Carolina; Los Angeles County, California; northern Manhattan, New York; and St. Paul, Minnesota, between 2000 and 2002 (Bild et al. 2002). These men and women were 45–84 years of age and free of clinical cardiovascular disease at baseline. The MESA and Coarse Particulate Matter (MESA Coarse) substudy, on which this analysis is based, included the 3,295 participants from Chicago, Forsyth County, and St. Paul. These areas were selected for intensive air pollution sampling and modeling of PM_10–2.5_. Institutional review board approval at each site and written informed consent from each participant were obtained.

*Inflammation and coagulation biomarkers*. Biomarkers were measured in fasting blood collected at the MESA baseline examination (2000–2002). Seven biomarkers were considered: IL-6, CRP, fibrinogen, total homocysteine, factor VIII, D-dimer, and PAP. These were selected inflammation or coagulation markers that were measured on all participants. Samples were analyzed at the University of Vermont Laboratory for Clinical Biochemistry Research following rigorous quality control procedures, as reported elsewhere (Bild et al. 2002).

Briefly, IL-6 was measured using ultra-sensitive enzyme-linked immunosorbent assay (Quantikine HS Human IL-6 Immunoassay; R&D Systems, Minneapolis, MN) with a lower detection limit of < 0.094 pg/mL [coefficient of variation (CV): 6.3%]. CRP and fibrinogen were measured using the BNII nephelometer (N High Sensitivity CRP, N Antiserum to Human Fibrinogen; Dade Behring, Inc. Deerfield, IL). CRP intra-assay CVs ranged from 2.3 to 4.4% and interassay CVs ranged from 2.1 to 5.7%. Fibrinogen intra-assay and interassay CVs were 2.7 and 2.6%. Homocysteine was measured using a fluorescence polarization immunoassay (IMx Hcy assay; Axis Biochemicals ASA, Oslo, Norway) with the IMx analyzer (Abbott Diagnostics, Abbott Park, IL). Factor VIII coagulant activity was determined using the clot time in factor VIIIc deficient plasma and the presence of activators utilizing the Sta-R analyzer (STA-Deficient VIII; Diagnostica Stago, Parsippany, NJ). The results are expressed as percent factor VIII. D-dimer was measured by an immunoturbidometric method on the Sta-R analyzer (Liatest D-DI; Diagnostica Stago, Parsippany, NJ). A two-site enzyme-linked immunosorbent assay (ELISA) that uses two monoclonal antibodies was used to measure PAP (Holvoet et al. 1986).

Because past research suggests that a biomarker composite score is indicative of overall inflammation/coagulation burden (Jenny et al. 2010; Pollitt et al. 2008), we created two summary scores as our primary end points. These groupings were based on *a priori* scientific judgment and included IL-6, CRP, fibrinogen, and total homocysteine for inflammatory burden and D-dimer, factor VIII, and PAP for coagulation. Each score was created by summing the *z*-scores of each natural log-transformed biomarker, estimated as z*_i_* = (x*_i_* – μ)/σ, where x*_i_* is the measured level for person *i*, μ is the population mean, and σ is the population standard deviation. Alternative scorings derived from a principal component analysis (PCA) were also investigated and associations reported for individual log-transformed biomarkers.

*Air pollution*. As described elsewhere, we used land use regression spatial prediction models using project-specific measurements and geographic data (e.g., land use, vegetation, emissions) to estimate concentrations of PM_10–2.5_ mass and selected PM_10–2.5_ components for each participant (Zhang et al. 2014). On the basis of a modified positive matrix factorization analysis, we used copper as an indicator of brake wear, zinc of tire wear, phosphorus of agriculture, and silicon of soil and road dust (Sturtz et al. 2014). PM_10–2.5_ endotoxin concentrations were also investigated as an important innate immune modulating component of bacterial cell membranes (Haᵭina et al. 2008). All of our models were developed uniquely for each study site and had cross-validated *R*^2^ ranging from 0.3 to 0.9 (Zhang et al. 2014). Estimates of long-term concentrations of PM_2.5_ and light-absorbing carbon (LAC), an indicator of tailpipe emissions from motor vehicles, were also available from the Multi-Ethnic Study of Atherosclerosis and Air Pollution (MESA Air) spatiotemporal model (Kaufman et al. 2012; Szpiro et al. 2010). These pollutants were explored as potential confounders of relationships with PM_10–2.5_.

PM_10–2.5_ mass and components as well as LAC concentrations were averaged over the 5 years preceding a participant’s baseline exam. Although time was incorporated into our estimates using residential history, the spatial patterning of pollution was assumed to be constant over time, such that data from 2009 for PM_10–2.5_ and 2006–2009 for LAC reflect the patterns that would have been observed preceding the baseline examination in 2000–2002. This is supported by an unpublished analysis that demonstrated general spatial stability of pollution over multiple years in the states of interest. PM_2.5_ was predicted for the 1 year preceding baseline because the MESA Air models incorporated time but were limited by availability of pollution measurements before 1999. For all scenarios, concentrations are intended to represent long-term exposures.

*Covariates*. Participant data were obtained using standardized methods described elsewhere (Bild et al. 2002). Height, weight, systolic and diastolic blood pressure, creatinine, low-density lipoprotein (LDL) and high-density lipoprotein (HDL), and total cholesterol were all measured during the clinical examination. Personal characteristics including sex, age, race/ethnicity, marital status, employment, and education were collected via technician-administered questionnaire. These questionnaires further collected information on first-degree family history of heart attacks and stroke as well as health behaviors including exposures to cigarette smoke, alcohol consumption, and weekly physical activity level. Medication use was also recorded by study technicians. Disease status included diabetes as defined by measured fasting serum glucose levels and medication use consistent with the 2003 American Diabetes Association guidelines (Genuth et al. 2003) and hypertension as defined by a measured systolic blood pressure > 140 mm Hg, diastolic blood pressure > 90 mm Hg, or use of anti-hypertensive medications. To capture a participant’s contextual environment, a neighborhood socioeconomic score (NSES) was derived for the baseline address using census tract–level data on education, occupation, median home values, and median household income from the 2000 Census (Hajat et al. 2013).

*Statistical analysis*. All statistical modeling for this paper was conducted in SAS v9.3 (SAS Institute Inc., Cary, NC). Before analysis, multiple imputation through chained equations was used to impute values for the 821 of 3,295 participants with missing exposure (16%), outcome (5%), or covariate information (6%) (Raghunathan et al. 2001). Twelve imputed data sets were generated, each after 10 iterations, using IVEware v0.2 (University of Michigan Institute for Social Research, Ann Arbor, MI). Our imputation model included variables in our primary analytic model as well as numerous auxiliary variables (e.g., triglycerides, total cholesterol, fasting glucose, and other medical conditions). The impact of imputation was assessed by examining imputed and non-imputed values, comparing with a complete-case analysis, and exploring the sensitivity of imputation models.

Multivariable regression models were used to estimate associations and adjust for confounders. Because multiple imputation was used, our confidence limits were adjusted to reflect the added uncertainty through Rubin’s rules (Rubin 1987). Models were staged to examine the sensitivity to potential confounders, including some that may also be a consequence of air pollution and/or inflammation and coagulation. Model 1 included demographic variables: age, sex, and race/ethnicity. Model 2 added socioeconomic factors (NSES, current employment, current marital status, education, household size, and home ownership) and behavioral factors (alcohol consumption, active and passive smoke exposure, physical activity). Model 3 added health status parameters that might be confounders or a downstream consequence of exposure and/or our outcomes [diabetes, hypertension, family history of heart attack, body mass index (BMI), systolic blood pressure (SBP), diastolic blood pressure (DBP), hypertension, HDL, creatinine, nonsteroidal anti-inflammatory drugs, steroids, aspirin, oral anti-inflammatory asthma drugs, and anti-hypertensives]. Finally, model 4 added adjustment for metropolitan area, which may reduce bias from potential confounding but may also reduce power by controlling for between-area differences in exposure variability. Age, NSES, BMI, SBP, DBP, HDL, and creatinine were modeled as continuous terms, whereas race/ethnicity (non-Hispanic white, non-Hispanic black, Hispanic, Chinese), education (high school or less, some college, associates/bachelors degree, graduate degree), exercise (tertiles of physical activity per day), household size (1, 2, 3–4, ≥ 5), active and passive smoke exposure (never smoker/no passive smoke, never smoker/passive smoke, former smoker/no passive smoke, former smoker/passive smoke, current smoker), alcohol consumption (never, former, current), and site (Winston-Salem, Chicago, St. Paul) were modeled as multicategory variables. Sex, current marital status, current employment status, home ownership, hypertension, anti-inflammatory medication, anti-hypertensive use, diabetes, and family history of were modeled as binary variables. All associations were scaled to each pollutant’s interquartile range (IQR) and reported with their 95% confidence intervals (CIs) (α = 0.05).

Heterogeneity of association with each exposure was assessed by including interaction terms in model 4 with sex, age, race/ethnicity, metropolitan area, obesity, diabetes, and metabolic syndrome. Additional sensitivity analyses included restriction to those who had not moved residence within 5 years, those without an infection in the previous 2 weeks, and former or never smokers. Further control for meteorology (temperature and relative humidity on the day of the examination), season of the examination, statins, and other pollutants (PM_2.5_, LAC) were also explored in order to investigate any potential confounding by these factors.

## Results

The mean age of the 3,295 MESA Coarse participants was 62 years, and 52% were female (Table 1). Non-Hispanic whites constituted 53% of the population, non-Hispanic blacks 24%, Hispanics 14%, and Chinese 9%. By design, Hispanic participants were present only in St. Paul, whereas Chinese participants were only in Chicago. In general, most participants had a college degree or advanced degree (55%) and were not current smokers (87%). Measures of coagulation were generally similar across metropolitan areas, though inflammatory markers were generally lower in Chicago than the other two areas.

**Table 1:** Descriptive statistics [mean ± SD, percent, or geometric mean (GSD)] for MESA Coarse population.

Characteristic	Percent missing	All	Winston-Salem	St. Paul	Chicago
*n*		3,295	1,072	1,060	1,163
Age (years)	0.0	62 ± 10	63 ± 10	60 ± 10	62 ± 10
Female (%)	0.0	52	53	51	53
Race/ethnicity (%)	0.0
White		53	53	57	48
Chinese		9	0	0	26
Black		24	46	0	26
Hispanic		14	0	43	0
Income (%)	5.2
< $20,000		17	14	23	14
$20,000–$30,000		12	11	15	9
$30,000–$40,000		12	13	15	7
$40,000–$50,000		11	11	13	8
$50,000–$75,000		21	26	19	18
> $75,000		29	26	15	44
Married (%)	0.0	63	68	59	63
Education level (%)	0.1
High school or less		28	29	41	15
High school and some college		17	18	16	16
College		33	36	32	33
Advanced degree		22	18	10	36
Alcohol (%)	0.4
Never		16	20	11	17
Former		23	31	24	14
Current		61	50	65	69
Smoking status (%)	0.1
Never		47	44	44	52
Former		40	42	40	38
Current		14	14	16	11
Diabetes (%)	0.3	11	12	12	9
BMI (kg/m^2^)	0.0	28 ± 5	29 ± 5	29 ± 5	27 ± 5
Coarse particles
PM_10–2.5_ (μg/m^3^)	8.4	5.0 ± 1.7	3.8 ± 1.3	5.5 ± 1.9	5.6 ± 1.2
Cu (ng/m^3^)	8.8	4.5 ± 2.7	2.5 ± 1.0	3.6 ± 1.1	7.4 ± 2.3
Zn (ng/m^3^)	10.1	9.9 ± 10.8	3.4 ± 3.7	5.5 ± 3.9	19.8 ± 12.1
P (ng/m^3^)	11.5	16.3 ± 3.7	19.9 ± 2.4	13.1 ± 2.4	15.8 ± 2.9
Si (μg/m^3^)	9.9	0.4 ± 0.1	0.4 ± 0.1	0.5 ± 0.1	0.4 ± 0.1
Endotoxin (EU/m^3^)	7.0	0.1 ± 0.1	0.0 ± 0.1	0.1 ± 0.0	0.0 ± 0.0
Fine particles
PM_2.5_ (μg/m^3^)	5.6	14.6 ± 2.2	15.4 ± 0.9	12.1 ± 1.5	16.2 ± 1.5
LAC (10^–5^/m)	6.6	0.5 ± 0.1	0.5 ± 0.1	0.5 ± 0.1	0.6 ± 0.1
Inflammation markers
Inflammation score	3.2	0.0 ± 2.7	0.2 ± 2.7	0.4 ± 2.6	–0.5 ± 2.7
IL-6 (pg/mL)	2.9	1.2 (2.0)	1.3 (1.9)	1.4 (1.9)	1.1 (2.0)
CRP (mg/L)	0.8	1.9 (3.2)	2.3 (3.2)	2.2 (3.0)	1.5 (3.2)
Fibrinogen antigen (mg/dL)	0.6	334.8 (1.2)	331.0 (1.2)	344.4 (1.2)	329.8 (1.2)
Total homocysteine (μmol/L)	0.2	8.9 (1.4)	9.0 (1.4)	8.9 (1.4)	8.9 (1.3)
Coagulation markers
Coagulation score	2.8	0.0 ± 2.1	0.1 ± 2.1	–0.1 ± 2.1	0.0 ± 2.2
Factor VIII (%)	0.5	146.3 (1.5)	147.8 (1.5)	143.4 (1.5)	147.6 (1.5)
D-Dimer (μg/mL)	0.6	0.2 (2.6)	0.2 (2.6)	0.2 (2.4)	0.2 (2.6)
PAP (nM)	2.6	4.4 (1.5)	4.5 (1.5)	4.3 (1.5)	4.5 (1.5)

The overall mean PM_10–2.5_ mass concentration was 5.0 ± 1.7 μg/m^3^ with slightly higher levels in Chicago (5.6 ± 1.2 μg/m^3^) and lower levels in Winston-Salem (3.8 ± 1.3 μg/m^3^) (Table 1). More noticeable differences were observed between sites with respect to chemical species, with Chicago exhibiting higher concentrations of traffic-related pollutants, as indicated by copper, zinc, and LAC. In contrast, Winston-Salem had the lowest concentrations of copper and zinc but the highest concentrations of phosphorus, a tracer of soil treated with fertilizer. St. Paul had the highest levels of endotoxin. While the different chemical components of PM_10–2.5_ were highly correlated in Chicago (0.5–0.8), modest correlations were observed in St. Paul (0.2–0.6), and generally low correlations were observed in Winston-Salem (0.0–0.5) (see Supplemental Material, Table S1). Endotoxin concentrations were very low (Table 1) and were generally weakly and negatively correlated with PM_10–2.5_ mass and constituents (–0.4 to 0.2) (see Supplemental Material, Table S1).

Markers of inflammation showed some differences by study site with evidence of lower crude levels of IL-6, CRP, and fibrinogen in Chicago than the other locations (Table 1). In contrast, slightly lower mean levels of factor VIII and PAP were observed in St. Paul compared with other locations. Correlations between the different markers were generally low, though there was some shared variance between IL-6, CRP, and fibrinogen—three of our *a priori* selected inflammatory biomarkers (*r* = 0.4–0.5) (see Supplemental Material, Table S2). This shared variance was also reflected in a PCA, where the same three biomarkers loaded highly (> 0.4) on the same factor (results not shown).

**Figure 1:**
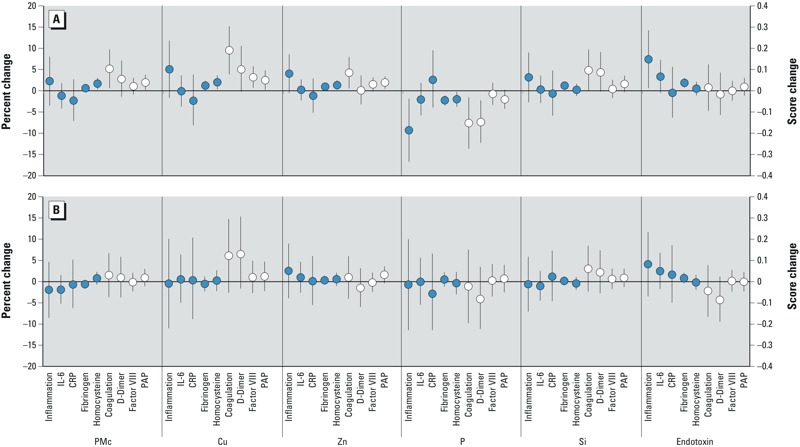
Associations between long-term exposures to PM_10–2.5_ mass and components with biomarkers of inflammation and coagulation before (*A*) and after (*B*) adjustment for study site. Abbreviations: Cu, copper; P, phosphorus; PMc, PM_10–2.5_ mass; Si, silicon; Zn, zinc. Associations with indicators of inflammation are in blue, whereas indicators of coagulation are in white. Inflammation and coagulation scores on the secondary axis, and all other biomarkers are reported as a percent change. All associations were adjusted for age, sex, race, city, marital status, education, employment, household size, home ownership, NSES, alcohol consumption, active and passive smoke, physical activity, family history of stroke or heart attack, BMI, blood pressure, cholesterol, creatinine, diabetes, and anti-inflammatory and blood pressure medications. Associations are scaled to IQRs of 2 and 0.1 μg/m^3^ for PM_10–2.5_ and silicon and 4, 11, and 6 ng/m^3^ for copper, zinc, and phosphorous, respectively. Endotoxin is scaled to 0.08 EU/m^3^.

**Figure 2:**
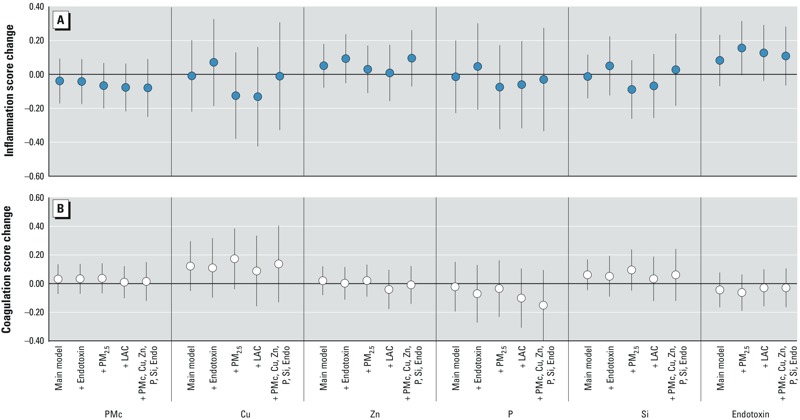
Associations between long-term exposures to PM_10–2.5_ mass and components and an inflammation (*A*) and coagulation (*B*) score adjusted for co-pollutants. Abbreviations: Cu, copper; Endo, endotoxin; LAC, light-absorbing carbon; P, phosphorus; PMc, PM_10–2.5_ mass; Si, silicon; Zn, zinc. All associations were adjusted for age, sex, race, city, marital status, education, employment, household size, home ownership, NSES, alcohol consumption, active and passive smoke, physical activity, family history of stroke or heart attack, BMI, blood pressure, cholesterol, creatinine, diabetes, and anti-inflammatory and blood pressure medications. Associations are scaled to IQRs of 2 and 0.1 μg/m^3^ for PM_10–2.5_ and silicon and 4, 11, and 6 ng/m^3^ for copper, zinc, and phosphorous, respectively. Endotoxin is scaled to 0.08 EU/m^3^.

*Associations with inflammatory markers*. After adjustment for demographics, socioeconomic, behavioral, and health factors, we found limited evidence for relationships between markers of inflammation and PM_10–2.5_ or its components. Before adjustment by location, the strongest evidence of an association was observed for endotoxin, with a 0.15-unit higher inflammation score per 0.08 EU/m^3^ (95% CI: 0.01, 0.28; *p* = 0.03) (Figure 1A). Positive associations were also found for the inflammation score with PM_10–2.5_ mass, copper, zinc, and silicon, though these associations were imprecise and failed to meet statistical significance. A negative association was observed between the inflammation score and phosphorus. After control for study site, only associations with endotoxin, and to a lesser extent zinc, remained with the inflammation score (Figure 1B). These associations were weaker in magnitude and had inflated standard errors. Site-adjusted associations of inflammation score with endotoxin and zinc were, however, robust to control for other PM_10–2.5_ components and stronger when adjusted for PM_2.5_ and LAC in two-pollutant models (Figure 2A). PM_2.5_ and LAC were themselves also independently associated with higher inflammation (0.39 per 3.8 μg/m^3^; 95% CI: 0.15, 0.63; *p* = 0.001 and 0.14 per 0.2 10^–5^/m; 95% CI: –0.07, 0.36; *p* = 0.18, respectively) in single-pollutant models with the same covariate adjustment (see Supplemental Figure S1). Sensitivity analysis using a PCA-derived inflammation score had qualitatively similar conclusions with the strongest relationships with endotoxin that demonstrated higher inflammation with higher endotoxin concentrations. As in our main analyses, associations with the PCA–derived inflammation score were weakened and less precise after control for study site (see Supplemental Material, Figure S2).

*Associations with coagulation markers*. Before adjustment for location, 2 μg/m^3^, 4 ng/m^3^, 11 ng/m^3^, and 0.1 μg/m^3^ higher levels of PM_10–2.5,_ copper, zinc, and silicon were associated with 0.10 (95% CI: 0.012, 0.20; *p* = 0.02), 0.19 (95% CI: 0.078, 0.30; *p* = 0.0008), 0.085 (95% CI: 0.01, 0.16; *p* = 0.02), and 0.10 (95% CI: –0.001, 0.19; *p* = 0.05) unit higher coagulation scores, respectively (Figure 1A). In contrast, higher phosphorus levels were associated with lower coagulation scores (–0.15 per 6 ng/m^3^; 95% CI: –0.27, –0.031; *p* = 0.01). After control for location, however, only copper (0.12 per 4 ng/m^3^; 95% CI: –0.05, 0.30; *p* = 0.16) and silicon (0.06 per 0.1 μg/m^3^; 95% CI: –0.05, 0.17; *p* = 0.26) maintained consistent associations with the coagulation score, though these associations were weaker and had wider confidence intervals (Figure 1B). Relationships with the coagulation score (both with and without adjustment for study site) were driven by the component D-dimer (Figure 1) and were robust to adjustment for other pollutants (Figure 2). This is consistent with our finding of no statistically significant associations between PM_2.5_ or LAC and the summary coagulation score after site adjustment (see Supplemental Figure S1).

**Figure 3:**
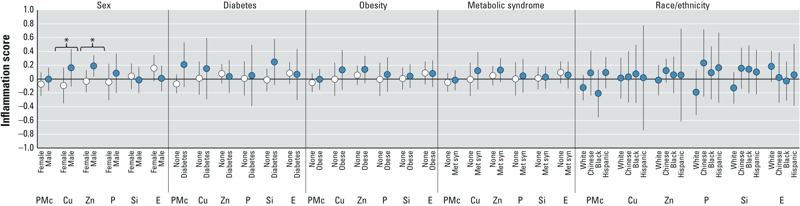
Associations between PM_10–2.5_ mass and components with inflammation score by sex, diabetes, obesity, metabolic syndrome, and race/ethnicity. Significant interactions are noted by asterisk (*). Abbreviations: Cu, copper; E, endotoxin; P, phosphorus; PMc, PM_10–2.5_ mass; Si, silicon; Zn, zinc. All associations were adjusted for age, sex, race, city, marital status, education, employment, household size, home ownership, NSES, alcohol consumption, active and passive smoke, physical activity, family history of stroke or heart attack, BMI, blood pressure, cholesterol, creatinine, diabetes, and anti-inflammatory and blood pressure medications. Associations are scaled to IQRs of 2 and 0.1 μg/m^3^ for PM_10–2.5_ and silicon and 4, 11, and 6 ng/m^3^ for copper, zinc, and phosphorous, respectively. Endotoxin is scaled to 0.08 EU/m^3^.

*Effect modification and sensitivity analyses*. Interaction terms were largely nonsignificant, though there was evidence of differences by sex (interaction *p*-value for copper = 0.03, zinc = 0.006) for inflammation scores. Specifically, we found positive associations scores among men (Cu: 0.16 per 4 ng/m^3^; 95% CI: –0.11, 0.44; Zn: 0.19 per 11 ng/m^3^; 95% CI: 0.03, 0.34) but no associations among women (Cu: –0.09 per 4 ng/m^3^; 95% CI: –0.35, 0.17; Zn: –0.03 per 11 ng/m^3^; 95%CI: –0.19, 0.13) (Figure 3). No significant effect modification was noted for coagulation (results not shown). Our conclusions in terms of directionality and significance remained robust to modeling of an inflammatory score derived by PCA (see Supplemental Material, Figure S2), additional control for weather, seasonality, statin therapy, exclusion of possible intermediates in the model, as well as the exclusion of persons with missing information (*n* = 821), current smokers (*n* = 446), and persons living in their neighborhood < 5 years (*n* = 594) (results not shown).

## Discussion

In a large, multicenter cohort we found suggestive, but inconclusive, evidence that higher long-term PM_10–2.5_ concentrations were associated with greater inflammation and coagulation. Associations of endotoxin with the inflammation score and copper with the coagulation score were the strongest and most robust to control for study site. Relationships with these summary scores were driven by IL-6 and D-dimer, respectively. These associations were further robust to control for other components of PM_10–2.5_ as well as PM_2.5_ and LAC, which were also associated with markers of inflammation. Overall, our results suggest that long-term PM_10–2.5_ exposures may be related to higher inflammation and coagulation, but the magnitude of the association appeared to depend on particle composition. Given the modest significance of our findings, further research is needed to confirm these suggestive associations.

This study adds to the extremely limited epidemiology literature on PM_10–2.5_, especially with respect to long-term exposures. By investigating PM_10–2.5_ components, including endotoxin, we were furthermore able to explore indicators of different sources of PM_10–2.5_ while controlling for the important co-pollutants PM_2.5_ and LAC. Although our overall results were inconclusive, we estimated that a 0.08-EU/m^3^ larger 5-year average endotoxin concentration was associated with a 0.15 (95% CI: 0.01, 0.28; *p* = 0.03) and 0.08 (95% CI: –0.07, 0.23; *p* = 0.28) unit higher inflammation score before and after control for location, respectively. Similarly, a 4-ng/m^3^ larger copper concentration was associated with a 0.19 (95% CI: 0.08, 0.30; *p* = 0.0008) and 0.12 (95% CI: –0.05, 0.30; *p* = 0.16) unit higher coagulation score without and with control for location, respectively. Using associations with age from our main models (0.06 unit of inflammation score/year of age; 95% CI: 0.05, 0.07, and 0.07 unit coagulation score/year of age; 95% CI: 0.06, 0.08), we estimate that these differences are roughly equivalent to the differences in inflammation and coagulation scores between people approximately 1–3 years apart in age. Although control for location reduced the magnitude of our associations, it also widened our confidence intervals, suggesting that control for confounding by location may have also overcontrolled for exposure by eliminating variability between metropolitan areas.

Although very few epidemiological investigations have explored long-term associations between particulate matter and inflammation, higher annual average concentrations of PM_10_ have been associated with greater white blood cell counts in a cross-sectional analysis of American adults in the National Health and Nutrition Examination Survey (Chen and Schwartz 2008). Greater long-term exposures to PM_2.5_ were also cross-sectionally associated with larger CRP and fibrinogen levels in male, but not female, German adults (*n* = 4,032) of the Heinz Nixdorf Recall Study (Hoffmann et al. 2009). In three cross-sectional samples of the British population, however, no associations were found for CRP and fibrinogen with annual PM_10_ levels (Forbes et al. 2009). Inconsistencies among these large studies could reflect geographic and temporal differences in the composition of air pollution.

In this study, we were able to explore more than simply mass, and found the strongest associations for coagulation and inflammation with copper and endotoxin, respectively. These associations are consistent with numerous experimental studies which have documented increased inflammatory markers *in vivo* and *in vitro* with short-term exposures to endotoxin (Monn and Becker 1999; Schwartz et al. 1994; Soukup and Becker 2001; Thorne et al. 2005) and transition metals (Gerlofs-Nijland et al. 2009; Lippmann and Chen 2009). In fact, PM_10–2.5_ copper has previously been linked to inflammation in mice and alveolitis and leukocytes in the lungs of rats (Gerlofs-Nijland et al. 2007; Happo et al. 2010).

The patterning of associations with copper, an indicator of brake wear, and to a lesser extent zinc, an indicator of tire wear, and silicon, a correlate of road dust, may also suggest health impacts of PM_10–2.5_ from traffic. This may be attributable to the metals themselves or other correlated pollutants. Because our findings for copper and silicon were relatively robust to control for PM_2.5_ mass and LAC, road dust may have associations independent of primary combustion-related traffic pollution. This is consistent with past research, which has documented associations between freshly generated brake wear emissions and oxidative stress and inflammation in human lung cells, macrophage-mediated inflammation and PM_10_ from tire wear in mice, and enhanced cytokine production in human and rat cells with mineral-rich PM_10_ collected from roadways (Gasser et al. 2009; Hetland et al. 2004; Mantecca et al. 2009, 2010). Research from the Netherlands has also shown that PM_10–2.5_ from locations with high levels of stop-and-go traffic was associated with higher levels of tumor necrosis factor-α (TNF-α) *in vitro* whereas altered macrophage activity was associated with PM_10–2.5_ from locations with free-flowing but not stop-and-go traffic (Steenhof et al. 2013).

Counter to our hypothesis, we found stronger associations for inflammation with PM_2.5_ mass than with PM_10–2.5_ mass. Though PM_10–2.5_ has not been well studied in humans previously, it has been suggested that this size fraction may have greater inflammatory potential due to high levels of endotoxin, compared to PM_2.5_. Support for this hypothesis comes from mechanistic *in vitro* and *in vivo* studies that examined cytokine production after blocking specific bacterial recognition pathways. For example, one *in vitro* study found an attenuated IL-6 cytokine association with PM in human alveolar macrophages after using CD14 antibodies to inhibit bacterial recognition (Becker et al. 2002). Another study identified diffential roles for Toll-like receptors 2 and 4 in the stimulation of IL-6 and TNF-α from exposures to bacterial cell walls using knockout mice (Takeuchi et al. 1999). In addition, inflammation from PM_10–2.5_ has been shown to be attenuated by endotoxin inhibitors or heat activation as measured by lower macrophage mRNA (messenger RNA) TNF-α content of induced sputum in human volunteers (Alexis et al. 2006) and lower observed levels of IL-6 in human cell lines (Becker et al. 2002; Monn and Becker 1999; Soukup and Becker 2001). This attenuation was not observed in mice (Wegesser and Last 2008) nor for all inflammatory markers (Alexis et al. 2006), however. Although we found associations between endotoxin and inflammation, our weak associations with PM_10–2.5_ may be attributable to low levels of endotoxin. Throughout the three cities, our median ambient concentration was 0.07 EU/m^3^ (maximum, 0.48 EU/m^3^). Similar levels were reported for two small German towns, but concentrations were nearly 10 times lower than median levels in PM_10_ across 13 California communities and three times lower than levels in Los Angeles (Heinrich et al. 2003; Mueller-Anneling et al. 2004). Our levels are, however, higher than the 0.015-EU/m^3^ concentrations measured in PM_2.5_ from Munich (Carty et al. 2003). Other possible explanations for weak associations could be overcontrol for factors that predict indoor endotoxin levels, including poverty and education, the lack of indoor or personal data, which are more strongly related to inflammation (Thorne et al. 2009), or these findings could simply be attributable to chance.

Other weaknesses of this investigation include misalignment of the exposure and outcome data and a lack of temporal resolution that prohibits us from exploring short-term exposures and critical time–exposure windows. This analysis also does not account for differing measurement error by pollutant. This may be important when comparing the relative strength of different components, so caution is prudent when interpreting the results of studies such as this one. Finally, blood may not be the most sensitive medium to detect associations with pollutants such as endotoxin if it is not sufficiently proximal to the tissues where inflammation and coagulation may occur. As an example, one human study documented associations between PM and inflammatory markers using nasal lavage but not blood even at levels between 0.5 and 26.2 EU/m^3^ (Steenhof et al. 2013).

This study adds to the literature by investigating long-term associations between inflammation and individual-level estimates of PM_10–2.5_ mass, components, and endotoxin. The use of summary scores for inflammation and coagulation is also a new approach for exploring the impacts of air pollution. The benefit of this approach is that it treats the inflammatory and coagulation processes as a summary of several concurrent pathways, which may be helpful when there are shared mechanisms at play. The obvious drawback is that it pools findings across biomarkers with equal weighting. Nevertheless, sensitivity analysis demonstrated consistent associations with an inflammatory score derived by PCA. In addition, this approach has been used previously in MESA and other investigations to characterize overall inflammatory burden with associations reported with outcomes including functional decline, arterial stiffness, hospitalization, and death (Reuben et al. 2002; Salanitro et al. 2012; van Bussel et al. 2011).

## Conclusion

This research suggests that persons with higher long-term exposures to PM_10–2.5_ may have higher inflammation and coagulation than others, though our findings were ultimately inconclusive. Because we saw a patterning of association with PM_10–2.5_ originating from traffic (i.e., copper, zinc, and silicon) and biological material (i.e., endotoxin), these results may indicate that the magnitude of an inflammatory response to PM_10–2.5_ in humans depends on particle composition.

## Supplemental Material

(542 KB) PDF
