# Supplementary material for: Markers of Inflammation and Coagulation after Long-Term Exposure to Coarse Particulate Matter: A Cross-Sectional Analysis from the Multi-Ethnic Study of Atherosclerosis
Source: Environ Health Perspect. 2015 Jan 20;123(6):541–8. doi: 10.1289/ehp.1308069 (PMC4455582; doi:10.1289/ehp.1308069)
Supplement: (542 KB) PDF [file ehp.1308069.s001.508.pdf]

Environmental Health Perspectives doi:10.1289/ehp.1308069

Supplemental Material

Markers of Inflammation and Coagulation after Long-Term Exposure to Coarse Particulate Matter: A Cross-Sectional Analysis from the Multi-Ethnic Study of Atherosclerosis

Sara D. Adar, Jennifer D'Souza, Kari Mendelsohn-Victor, David R. Jacobs Jr., Mary Cushman, Lianne Sheppard, Peter S. Thorne, Gregory L. Burke, Martha L. Daviglus, Adam A. Szpiro, Ana V. Diez Roux, Joel D. Kaufman, and Timothy V. Larson

Correlations of $PM_{10-2.5}$ mass and components are presented below in Table S1, overall and by study site. While the different chemical components of $PM_{10-2.5}$ were highly correlated in Chicago (rho: 0.5 to 0.8), modest correlations were observed in St Paul (rho: 0.2 to 0.6), and generally low correlations were observed in Winston Salem (rho: 0.0 to 0.5). Endotoxin was generally weakly and negatively correlated with $PM_{10-2.5}$ mass and species.

Table S1. Correlation matrix between exposure estimates.

**Exposure**	**$PM_{10-2.5}$**	**Cu**	**Zn**	**Ph**	**Si**	**Endotox**	**$PM_{2.5}$**	**LAC**	**NO_2**	**NO_x**
**Overall**										
$PM_{10-2.5}$	1	0.52	0.45	-0.06	0.54	0.17	0.00	0.37	0.49	0.44
Cu	.	1	0.78	0.03	0.21	-0.27	0.45	0.78	0.89	0.91
Zn	.	.	1	0.11	0.14	-0.24	0.42	0.81	0.75	0.80
Ph	.	.	.	1	-0.04	-0.45	0.52	0.27	-0.08	0.08
Si	.	.	.	.	1	0.22	-0.27	0.07	0.20	0.12
Endotoxin	.	.	.	.	.	1	-0.59	-0.38	-0.23	-0.34
$PM_{2.5}$	.	.	.	.	.	.	1	0.66	0.49	0.60
LAC	.	.	.	.	.	.	.	1	0.81	0.89
NO_2	.	.	.	.	.	.	.	.	1	0.95
NO_x	.	.	.	.	.	.	.	.	.	1
**Chicago**										
$PM_{10-2.5}$	1	0.75	0.71	0.76	0.81	-0.14	0.51	0.78	0.75	0.84
Cu	.	1	0.56	0.72	0.75	-0.25	0.46	0.68	0.74	0.85
Zn	.	.	1	0.64	0.54	-0.09	0.32	0.87	0.56	0.71
Ph	.	.	.	1	0.79	-0.35	0.58	0.72	0.64	0.77
Si	.	.	.	.	1	-0.35	0.58	0.71	0.73	0.81
Endotoxin	.	.	.	.	.	1	-0.31	-0.15	-0.22	-0.23
$PM_{2.5}$	.	.	.	.	.	.	1	0.46	0.59	0.61
LAC	.	.	.	.	.	.	.	1	0.70	0.85
NO_2	.	.	.	.	.	.	.	.	1	0.88
NO_x	.	.	.	.	.	.	.	.	.	1
**St.Paul**										
$PM_{10-2.5}$	1	0.51	0.22	0.23	0.45	0.14	0.17	0.35	0.36	0.36
Cu	.	1	0.50	0.44	0.50	-0.03	0.36	0.65	0.63	0.71
Zn	.	.	1	0.36	0.36	-0.05	0.22	0.60	0.41	0.49
Ph	.	.	.	1	0.56	0.05	0.19	0.29	0.26	0.33
Si	.	.	.	.	1	-0.14	0.32	0.44	0.40	0.41
Endotoxin	.	.	.	.	.	1	-0.05	-0.01	-0.04	-0.05
$PM_{2.5}$	.	.	.	.	.	.	1	0.55	0.74	0.65
LAC	.	.	.	.	.	.	.	1	0.76	0.80
NO_2	.	.	.	.	.	.	.	.	1	0.94
NO_x	.	.	.	.	.	.	.	.	.	1

Exposure	$PM_{10-2.5}$	Cu	Zn	Ph	Si	Endotox	$PM_{2.5}$	LAC	NO_2	NO_x
**Winston-Salem**										
$PM_{10-2.5}$	1	0.04	0.00	0.31	0.46	0.26	-0.30	-0.02	-0.11	-0.11
Cu	.	1	0.37	0.22	0.54	-0.28	0.42	0.61	0.67	0.66
Zn	.	.	1	0.21	0.27	-0.23	0.18	0.47	0.39	0.39
Ph	.	.	.	1	0.32	-0.16	-0.03	0.11	0.04	0.14
Si	.	.	.	.	1	-0.12	0.08	0.41	0.36	0.36
Endotoxin	.	.	.	.	.	1	-0.27	-0.22	-0.27	-0.32
$PM_{2.5}$	.	.	.	.	.	.	1	0.50	0.61	0.62
LAC	.	.	.	.	.	.	.	1	0.89	0.87
NO_2	.	.	.	.	.	.	.	.	1	0.90
NO_x	.	.	.	.	.	.	.	.	.	1

Correlations of the inflammatory and coagulation markers evaluated in this analysis are presented below in Table S2. Only modest correlations were observed between the markers with the strongest relationships between IL-6, CRP, and fibrinogen (rho: 0.4 to 0.5).

Table S2. Correlation matrix between inflammation and coagulation markers.

Marker	IL-6	CRP	Fibrinogen	Hcsyt	Factor-VIII	d-dimer	PAP
IL-6	1	0.53	0.44	0.18	0.19	0.30	0.17
CRP	.	1	0.48	0.00	0.15	0.26	0.16
Fibrinogen	.	.	1	0.08	0.24	0.30	0.38
Homocyt	.	.	.	1	0.08	0.15	0.16
Factor-VIII	.	.	.	.	1	0.22	0.20
d-dimer	.	.	.	.	.	1	0.36
Plasmin antiplasmin	.	.	.	.	.	.	1

Single pollutant models of the associations for $PM_{2.5}$ and LAC with the inflammatory and coagulatory markers are presented below in Table S2. These results indicate that $PM_{2.5}$ and LAC were each independently associated with higher inflammation in site-adjusted models (0.39 per 3.8 $\mu g/m^3$; 95% CI: 0.15, 0.63; p=0.001 and 0.14 per 0.2 10-5/m; 95% CI: -0.07, 0.36; p=0.18, respectively) (Figure S1).

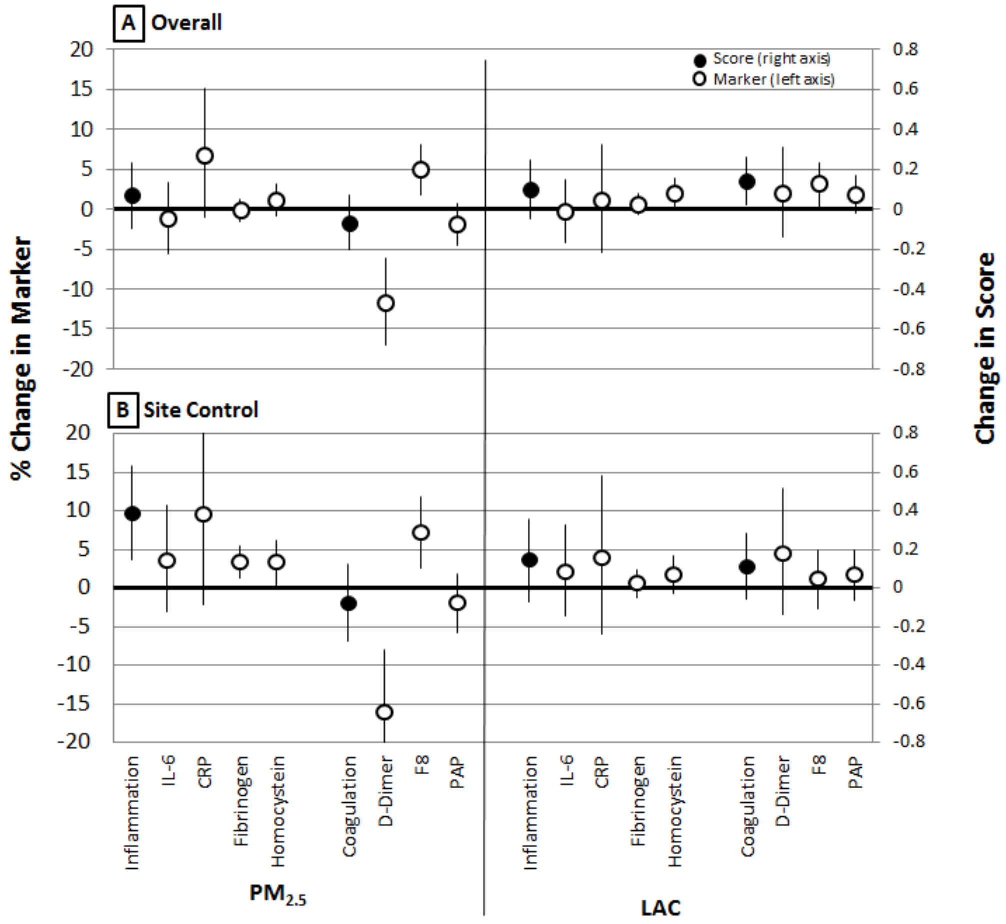

Figure S1. Associations between Long-Term Exposures to $PM_{2.5}$ Mass and LAC and an Inflammation and Coagulation (A) Before and (B) After Site Adjustment. All associations adjusted for age, gender, race, city, marital status, education, employment, household size, home ownership, NSES, alcohol consumption, active and passive smoke, physical activity, family history of stroke or heart attack, body mass index, blood pressure, cholesterol, creatinine, diabetes, and anti-inflammatory and blood pressure medications. Associations scaled to IQR of 3.8 $\mu g/m^3$ for $PM_{2.5}$ and 0,2 x 10^{-5}/m for LAC.

A comparison of results between our inflammation summary score derived by z-scores produced very similar results to an inflammation score derived using PCA. Again, the strongest relationships were identified with endotoxin. As in our main analyses, associations with the PCA-derived inflammation score were weakened and less precise after control for study site (Figure S2).

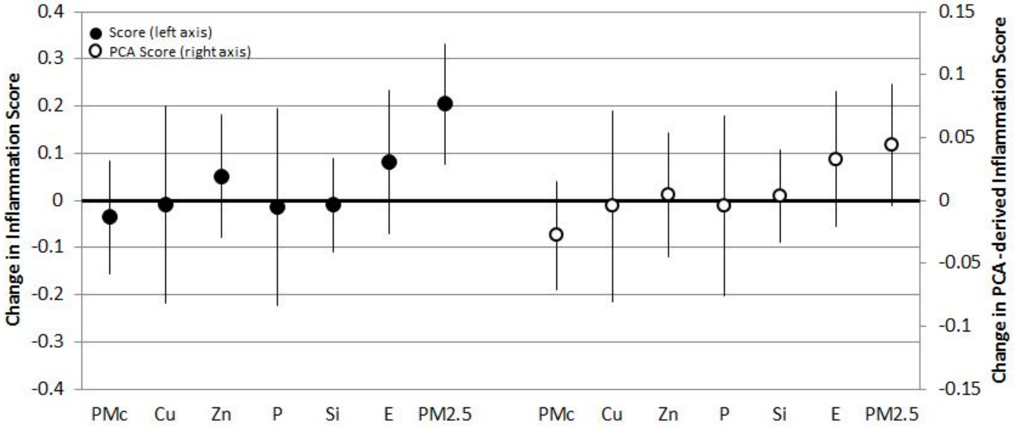

Figure S2. Site-Adjusted Associations between Long-Term Exposures to $PM_{10-2.5}$ Mass and Components with Inflammation Score Derived Using Z-Scores and PCA. PMc: $PM_{10-2.5}$ mass, Cu: copper, Zn: zinc, P: phosphorus, and Si: silicon. All associations adjusted for age, gender, race, city, marital status, education, employment, household size, home ownership, NSES, alcohol consumption, active and passive smoke, physical activity, family history of stroke or heart attack, body mass index, blood pressure, cholesterol, creatinine, diabetes, and anti-inflammatory and blood pressure medications. Associations scaled to IQR of 2 and 0.1 $\mu g/m^3$ for $PM_{10-2.5}$ and silicon and 4, 11, and 6 ng/m^3 for copper, zinc, and phosphorous, respectively. Endotoxin scaled to 0.08 EU/m^3.
